# Iron–Manganese–Magnesium Co-Modified Biochar Reduces Arsenic Mobility and Accumulation in a Pakchoi–Rice Rotation System

**DOI:** 10.3390/toxics14020112

**Published:** 2026-01-24

**Authors:** Jingnan Zhang, Meina Liang, Mushi Qiao, Qing Zhang, Xuehong Zhang, Dunqiu Wang

**Affiliations:** 1 College of Environmental Science and Engineering, Guilin University of Technology, Guilin 541004, China; zhangjingnan9@163.com (J.Z.); 17749621949@163.com (M.Q.); zhangqing@glut.edu.cn (Q.Z.); zhangxuehong@x263.net (X.Z.); wangdunqiu@sohu.com (D.W.); 2 Guangxi Key Laboratory of Environmental Pollution Control Theory and Technology, Guilin University of Technology, Guilin 541004, China; 3 Engineering Research Center of Watershed Protection and Green Development, Guilin University of Technology, Guilin 541006, China

**Keywords:** paddy soil remediation, arsenic contamination, food safety in rice, Fe-Mn-Mg modified biochar, pakchoi–rice rotation, arsenic bioavailability

## Abstract

Arsenic (As) contamination in paddy soils poses a serious risk to rice safety and human health. To mitigate this issue, we developed a low-temperature, partially pyrolyzed Fe/Mn/Mg-modified biochar (FMM-BC) and evaluated its performance and mechanisms for remediating As-contaminated soil through a pakchoi–rice rotation pot experiment, aiming to reduce As accumulation in rice grains and pakchoi. The results indicated that FMM-BC application altered soil physicochemical properties and As speciation, reducing both water-soluble and bioavailable As and promoting its transformation from exchangeable to more stable organic-bound and residual fractions. Compared with the control, FMM-BC application reduced arsenic content in rice stems, leaves, and brown rice to 1.94 mg∙kg^−1^, 5.24 mg∙kg^−1^, and 1.21 mg∙kg^−1^, respectively. In contrast, unmodified biochar (BC) increased As bioavailability and plant uptake, underscoring the importance of Fe/Mn/Mg modification. FMM-BC also enhanced the translocation of Fe, Mn, and Mg within rice plants, thereby modifying internal As transport dynamics and suppressing its accumulation in aboveground tissues. Under FMM-BC treatment, arsenic content in pakchoi stems and leaves decreased to 1.19 mg∙kg^−1^ (vs. 1.96 mg∙kg^−1^ in the control), and brown rice declined to 0.27 mg∙kg^−1^ (vs. 1.49 mg∙kg^−1^ in the control)—well below the national food safety threshold (0.35 mg∙kg^−1^). These findings demonstrate that FMM-BC effectively stabilizes As in contaminated soils and reduces its transfer to edible plant parts, with Fe/Mn/Mg playing a key role in enhancing As immobilization and limiting its mobility within the soil–plant system.

## 1. Introduction

Arsenic (As) is a ubiquitous metalloid that threatens global food security and human health. It is a common toxic element in agricultural soils and is classified as a priority Class-I pollutant in environmental protection standards. In paddy soils, arsenic occurs predominantly as arsenate (As(V); $H_{2}{\mathrm{AsO}}_{4}^{-}/{\mathrm{HAsO}}_{4}^{2-}$) under oxidizing conditions, whereas under flooded, anoxic conditions it is present mainly as arsenite (As(III); H_3_AsO_3_) [1]. Arsenite is more mobile and more toxic than arsenate, and is readily absorbed by crops and accumulates in grains, accumulating in edible grains [2]. Among cereals, rice is particularly notable for accumulating arsenic at concentrations far higher than other crops. More than 200 million people across 70 countries are exposed long-term to arsenic through consumption of arsenic-contaminated staple foods, especially rice [3]. In tropical and subtropical regions, where non-seafood-based diets predominate, rice serves as a staple food and represents the primary route of human dietary As exposure, particularly in many Asian countries [4,5]. For example, in rural areas of Bangladesh, the long-term use of As-contaminated groundwater for irrigation has led to widespread agricultural soil pollution, posing a serious threat to rice production. Studies have shown that approximately 35% of the country’s population exceeds the World Health Organization’s (WHO) previously recommended provisional tolerable daily intake (PTDI) for As (0.0021 mg∙kg^−1^ body weight) through dietary intake, mainly from rice [4]. As the world’s largest producer and consumer of rice, China also faces the challenge of arsenic contamination in its paddy fields. Research has shown that, in certain surveyed populations in China, rice consumption accounts for approximately 60% of dietary inorganic As intake [6]. Consequently, As pollution in paddy fields can enter the food chain through the soil–rice–human pathway, posing a serious threat to food safety. Developing cost-effective strategies to control the absorption and transport of As in the soil–rice system, thereby reducing its entry into the food chain, has become an urgent global environmental priority.

There are two main strategies for remediating soil heavy metal contamination: one is to remove heavy metals from the soil (e.g., leaching, phytoremediation), and the other is to reduce their bioavailability and mobility through immobilization. Soil immobilization techniques, which involve the application of chemical passivators, stabilize heavy metals in the soil and reduce their migration to environmental media and organisms. These techniques have attracted widespread attention due to their relatively simple operation and low cost [7]. Common passivators include lime, apatite, attapulgite, shell powder, sepiolite, crop residues, manure, and biochar [8]. Among these, biochar (BC) has shown great potential in the remediation of As-contaminated soils because of its large specific surface area, abundant pore structure, oxygen-containing functional groups, and environmentally friendly properties [9]. Studies have confirmed that biochar effectively reduces As bioavailability in soil and inhibits As accumulation in rice. This occurs through multiple mechanisms: electrostatic adsorption and ligand-exchange mediated by oxygen-containing functional groups on the biochar surface; co-precipitation with Fe/Al (hydr)oxides that nucleate on the biochar surface; and physical entrapment of arsenic within biochar pores [10]. However, the efficiency of As fixation with biochar alone is often limited. This is mainly because biochar is typically alkaline and has a negatively charged surface, which can enhance the mobility of negatively charged arsenate ions (As(V)) in the soil solution and may even increase their bioavailability to plants [11]. To improve As fixation efficiency, modifying biochar with other metals has emerged as an important research direction [12].

In recent years, the modification of biochar with metal (hydro)oxides to enhance its As adsorption and fixation capacity has attracted significant attention. Studies have shown that, compared with single-metal modifications, the use of multiple metals (such as Fe, Mn, and Mg) to co-modify biochar can substantially improve its As removal performance [13]. Iron oxides are well recognized for their strong affinity for arsenic through adsorption, surface complexation, co-precipitation, and redox-mediated processes [14,15,16]. Manganese oxides exhibit high oxidative potential and can promote the oxidation of As(III) to the less mobile As(V), potentially increasing subsequent retention [17,18]. Magnesium-containing phases have also been shown to contribute to arsenate retention, primarily through electrostatic interactions and the formation of positively charged surface sites under acidic to neutral conditions [19,20]. The coexistence of Fe, Mn, and Mg phases may therefore provide complementary adsorption and redox-related functions [21]. Nevertheless, the extent to which such synergistic effects operate in soil systems remains uncertain, particularly when the modified material is produced at relatively low pyrolysis temperatures.

Importantly, most existing studies on metal-modified biochar have focused on arsenic removal from aqueous systems, while considerably fewer investigations have examined their behavior in arsenic-contaminated soils and plant. In addition, the remediation performance and potential mechanisms of Fe–Mn–Mg co-modified biochar derived from low-temperature, partially pyrolyzed biomass have not been systematically evaluated. Such materials may better resemble incompletely burned residues that can realistically occur in agricultural environments. These knowledge gaps limit our understanding of the applicability and effectiveness of Fe–Mn–Mg co-modified biochar for reducing arsenic bioavailability and crop accumulation under soil conditions.

Available arsenic in soil is a key factor influencing its uptake and accumulation in rice. Its mobility and speciation are influenced by soil pH, redox potential, dissolved organic matter, and the abundance of reactive Fe and Al (hydr)oxides [19]. Changes in these factors alter the equilibrium between As(III) and As(V) and the sorption–desorption dynamics, thereby regulating arsenic availability to plant roots [22]. Therefore, this study aimed to synthesize FMM-BC and evaluate its performance under a pakchoi–rice rotation system, with the following specific objectives: (1) To examine the effects of FMM-BC application on soil bioavailable As concentrations and key physicochemical properties. (2) To determine the influence of FMM-BC on As uptake, translocation, and accumulation in edible organs during different growth stages of pakchoi and rice. (3) To explore the possible mechanisms contributing to arsenic immobilization following the application of FMM-BC in the soil–crop system. (4) To provide an initial evaluation of the potential applicability of FMM-BC in arsenic-contaminated agricultural soils, offering preliminary insight relevant to food safety and land-use management.

## 2. Materials and Methods

### 2.1. Soil

The soil used for the pot experiment was collected from arsenic-contaminated agricultural fields near a smelter in Dachang Town, Nandan County, Hechi City, Guangxi Province, China (24°51′21.49″ N, 107°35′59.46″ E). The top 20 cm of surface soil was sampled (five-point composite sampling; GB/T 36197-2018 [23]), air-dried, crushed, and passed through a 2 mm nylon sieve and thoroughly homogenized by repeated mixing in a polyethylene container. The main physicochemical properties were as follows: pH (H_2_O), 5.95; total As, 179.76 mg∙kg^−1^; and soil type, paddy soil. Detailed physical and chemical properties of the soil are provided in Table S1. In addition, pakchoi–rice rotation is the typical cultivation system in southern China [24], and its inclusion in this study reflects local agricultural conditions as documented in previous agronomic surveys.

### 2.2. Preparation of FMM-BC

The production procedure for FMM-BC followed our previous work [25]. Briefly, 10 g of 40-mesh sieved sugarcane bagasse was immersed in 100 mL of 5% KOH solution (prepared by dissolving 5 g KOH in 100 mL deionized water) for 24 h. Predefined volumes of 0.5 mol·L^−1^ MnSO_4_, 0.5 mol·L^−1^ MgCl_2_, and 40 mL of 1 mol·L^−1^ Fe(NO_3_)_3_ were then added, followed by stirring at 400 rpm and 25 °C for 30 min. The pH was adjusted to 10.0 with 5 mol·L^−1^ KOH, and stirring continued for an additional 30 min. After 24 h of settling, the supernatant was discarded. The residue was vacuum-filtered and washed repeatedly with deionized water until reaching neutral pH, then rinsed twice with deionized water and ethanol. The filter cake was dried at 80 °C, pyrolyzed in a tubular furnace, heated at 5 °C·min^−1^ to 300 °C and maintained for 1 h (Text S1), cooled, sieved through a 0.15 mm mesh, and stored.

A response surface optimization experiment was designed using the activator-to–sugarcane bagasse mass ratio, the Mn^2+^:Mg^2+^ molar ratio of the modifiers, and the pyrolysis temperature as independent variables, with soil bioavailable arsenic content (Y_1_) and biochar yield (Y_2_) as response variables. A three-factor, three-level experimental design based on the Box–Behnken methodology was employed to optimize the preparation conditions of the FMM-BC. The detailed experimental design of the response surface methodology is provided in Table S2.

### 2.3. Pot Experimental Design

The pot experiment was conducted in a greenhouse at Guilin University of Technology, Guilin City, Guangxi Province, China, to simulate common agricultural practices in As-affected regions. The experimental treatments included soil control (CK), soil + BC, and soil + FMM-BC, with each treatment replicated three times, for a total of nine pots. Each pot received 4 kg of air-dried soil (3.846 kg soil + 0.154 kg biochar, corresponding to a 4.0% biochar amendment) (Text S3, Figure S1) and was evenly mixed. The control group (CK) did not receive biochar. Basal fertilization consisted of 0.6 g urea, 0.6 g KH_2_PO_4_, and 0.4 g K_2_SO_4_ per pot, corresponding to an N:P_2_O_5_:K_2_O ratio of 0.15:0.15:0.10 g·kg^−1^. Fertilizers were dissolved in a nutrient solution and thoroughly mixed into the soil. Soil water content was adjusted to 70% (Texts S4 and S5) of the field water-holding capacity, and the soil was incubated for two weeks before planting. Pakchoi was cultivated first, followed by rice. Before cultivating rice in the same pots, plants and roots were removed to reduce soil As levels after harvesting pakchoi.

#### 2.3.1. Pakchoi Cultivation

Pakchoi (Brassica rapa chinensis group), a commonly cultivated leafy vegetable in Asia. Guilin local Jiusi pakchoi was used as the test crop and cultivated from October 2023 to December 2023. Seeds were purchased from the Guilin Nongfuxing Vegetable Planting Base, and were selected for uniform size and quality, soaked in water for 30 min, and 40 seeds were sown into each pre-treated pot. When the seedlings developed their third true leaf, 20 seedlings were thinned, and at the fourth leaf stage, 10 seedlings were retained per pot. Deionized water was applied daily according to soil water requirements, and the total growth period lasted 65 days.

#### 2.3.2. Rice Cultivation

Basal fertilizers were applied as described previously, and soil water content was maintained at 70% of field water-holding capacity during a two-week soil incubation period. Rice seeds were soaked in deionized water for 24 h, then placed in a light incubator (light intensity 0–25,000 lx) at a constant temperature of 25 ± 2 °C. After 24–36 h, the seeds were removed and transferred into disposable plastic cups filled with fine sand, submerged in water, and maintained in the incubator at 25 ± 2 °C. The seeds were watered twice daily, in the morning and evening. Once the seeds germinated, they were irrigated with the prepared nutrient solution. After 10 days of growth, uniformly developed seedlings (8–12 cm in height) were selected and transplanted into cultivation pots. The rice variety Xiang Early Indica 45 was transplanted on 13 August 2024, with six seedlings per pot, later thinned to three seedlings per pot according to growth conditions. Daily agricultural management followed conventional local practices and was consistent across all treatments. Throughout the growth period, rice was continuously flooded with 3–5 cm of water and cultivated for 105 days at temperatures of 25–35 °C and relative humidity of 50–70%.

### 2.4. Sampling and Analytical Methods

Soil and plant samples were collected at different growth stages for each crop. For pakchoi, soil samples were taken before sowing and at harvest, with mature plants harvested at 65 days. For rice, soil samples were collected before transplanting, at the tillering stage, and at maturity, with rice harvested at 105 days.

#### 2.4.1. Soil Sample Analysis

Soil samples were air-dried, ground, and prepared for analysis of physicochemical properties. Soil pH was measured using a pH meter E-201-C (Leici, Shanghai, China) with a soil-to-deionized water ratio of 1:2.5 (*w*/*v*) following HJ 962-2018 [26]. Soil electrical conductivity (EC) was determined using a conductivity meter DDS-801 (Leici, Shanghai, China) with a soil-to-water ratio of 1:5 according to HJ 802-2016 [27]. Soil dissolved organic carbon (DOC) was measured using a TOC analyzer Multi N/C 3100 (Analytik, Jena, Germany). Soil and water were mixed at a ratio of 1:5 and shaken at 25 °C and 200 r·min^−1^ for 4 h, followed by centrifugation and filtration prior to analysis. Soil available As was determined using the ${NaHCO}_{3}$ extraction method [28], with a soil-to-solution ratio of 1:10. The mixture was shaken in a water bath at 25 °C and 180 r·min^−1^ for 2 h, then centrifuged and filtered through a 0.45 μm membrane. Water-soluble As was extracted at the same soil-to-solution ratio (1:10) by shaking in a water bath at 25 °C and 180 r·min^−1^ for 3 h, followed by centrifugation and filtration through a 0.45 μm membrane Bound As fractions were extracted using the five-step sequential extraction procedure modified by Wenzel et al. [29]. Total soil As was measured following the national standard GB/T 22105.2-2008 [30]. The certified reference soil (GBW07405) was processed in the same manner to verify analytical accuracy. Concentrations of total As, available As, water-soluble As, and bound As in the soil were quantified using atomic fluorescence spectrometry AFS, SA-20 (JiTian, Beijing, China).

#### 2.4.2. Plant Sample Analysis

Pakchoi samples were separated into roots, stems, and leaves, while rice samples were divided into roots, stems, leaves, and brown grains. After complete removal of the plants and root systems and thorough cleaning with deionized water, plant tissues were sectioned and initially heated in an oven at 105 °C for 1 h to inactivate pigments, followed by drying at 80 °C to constant weight. All tissues were then ground into fine powder, and dry weights were recorded. Plant tissues were digested using a mixture of HNO_3_ and HClO_4_ (4:1, *v*/*v*) on a hot plate [31]. Total arsenic concentrations in all samples were determined by AFS, SA-20 (JiTian, Beijing, China).

#### 2.4.3. Characterization of Material

BC and FMM-BC were characterized using X-ray diffraction (XRD; Bruker-AXS D8 ADVANCE, Karlsruhe, Germany), Fourier-transform infrared spectroscopy (FTIR; NICOLET 6700, Waltham, MA, USA), and X-ray photoelectron spectroscopy (XPS; Thermo Scientific K-Alpha, Waltham, MA, USA) to investigate their fundamental properties and remediation mechanisms. For XPS analysis, the C 1s peak at 284.8 eV was used as a reference for charge correction. The morphology of the samples was analyzed by scanning electron microscope (Gemini SEM 360, Oberkochen, Germany).

### 2.5. Data Analysis

All experimental data were processed using Origin 2024 (SR1 10.1.0.178) and Excel 2016 (2508 Build 16.0.19127.20402) and one-way analysis of variance (ANOVA) was conducted using IBM SPSS Statistics 27.0.1. Prior to conducting one-way ANOVA, the experimental data were tested for normality using the Shapiro–Wilk test and for homogeneity of variances using Levene’s test, both performed in IBM SPSS Statistics 27.0.1. The results confirmed that the data met the assumptions of normality (*p* > 0.05) and homogeneity of variances (*p* > 0.05), ensuring the validity of the subsequent ANOVA results. Pearson correlation and redundancy analyses were performed to evaluate relationships among soil physicochemical properties, available arsenic, water-soluble arsenic, and arsenic fractions in the soil. Multiple comparisons and Pearson correlation analyses were conducted using IBM SPSS Statistics 27.0.1, while redundancy analysis was carried out with Canoco 5.0 software.

Each treatment group was analyzed in triplicate, and three blank controls were included concurrently for each experimental batch. The calibration curves were established using a mixed mineral element standard solution (purchased from the National Center for Nonferrous Metals and Electronic Materials Analysis and Testing, GSB 04-1767-2004) diluted to a series of gradient concentrations (R2 > 0.999). Certified reference materials for total arsenic in soil (GBW 07401) were obtained from the China Standard Materials Research Center, and the recovery rate of total arsenic ranged from 72.92% to 127.08%. The certified reference materials Citrus Leaf (GBW10020, Institute of Geophysical and Geochemical Exploration) and Standard Rice Flour (GBW(E)100357, Institute of Iron and Steel Research) were used for quality control during the analysis of cabbage and rice samples. The spiked recoveries of As ranged from 86.7% to 105.9%.

## 3. Results and Discussion

### 3.1. Preparation Optimization and Structural Characteristics of FMM-BC

#### 3.1.1. Optimization of FMM-BC Preparation by Response Surface Methodology

Based on the Design-Expert 13.0 software, a total of 17 experimental runs were generated using the Box–Behnken design, and the corresponding results are summarized in Table S3. The experimental data were fitted using multiple regression analysis, and the significance of the models was evaluated by analysis of variance (ANOVA), with detailed results presented in Text S2, Tables S4 and S5.

**Figure 1:**
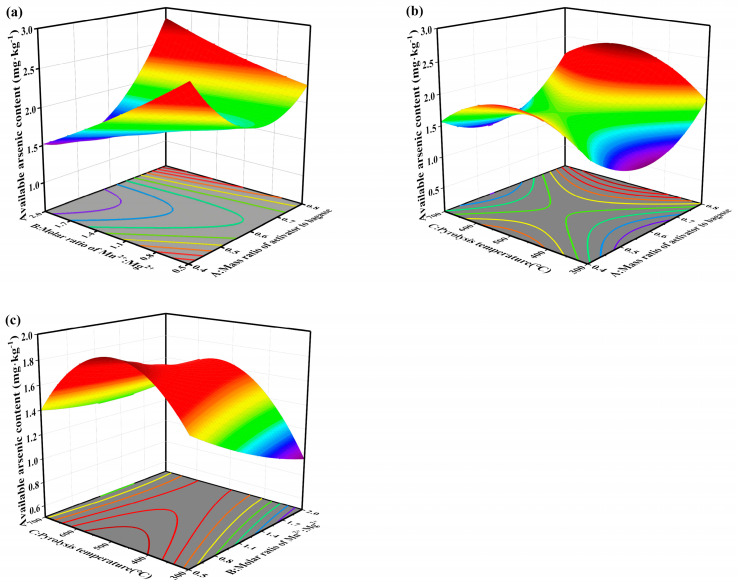
Influence of interaction of two factors on available arsenic contents. (**a**) response surface diagram of A and B; (**b**) response surface diagram of A and C; (**c**) response surface diagram of B and C (A: Mass ratio of activator to bagasse; B: Molar ratio of Mn^2+^:Mg^2+^; C: Pyrolysis temperature).

Three-dimensional response surface plots (Figure 1) further illustrated these interactions. Increasing the Mn^2+^:Mg^2+^ molar ratio at relatively low activator dosages significantly decreased available arsenic, whereas excessive activator addition weakened this effect, likely due to surface site saturation and residual salt accumulation. Pyrolysis temperature exhibited a non-linear effect, with available arsenic decreasing as temperature increased to approximately 300 °C, followed by a slight increase at higher temperatures. This suggests that moderate thermal treatment favors the formation of reactive metal (hydr)oxide phases and oxygen-containing functional groups, whereas excessive heating may reduce surface reactivity through partial structural collapse or functional group loss. Based on RSM optimization, the selected preparation conditions (activator-to-biomass ratio ≈ 0.6, Mn^2+^:Mg^2+^ = 2:1, and pyrolysis temperature = 300 °C) were adopted for subsequent experiments.

#### 3.1.2. Structural Characteristics of FMM-BC

**Figure 2:**
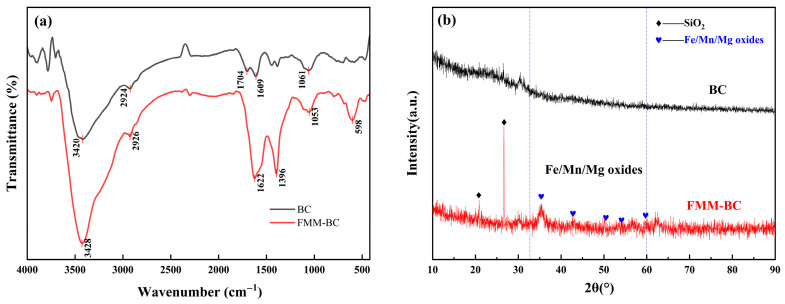
FTIR and XRD spectra of BC and FMM-BC: (**a**) FTIR spectra; (**b**) XRD spectra.

FTIR spectra (Figure 2a) showed that FMM-BC retained abundant oxygen-containing functional groups, including –OH (3420–3428 cm^−1^), –COOH (1396 cm^−1^), and –C=O (1760–1400 cm^−1^), which are largely associated with residual cellulose and hemicellulose generated under low-temperature pyrolysis. Absorption bands at 2924–2926 cm^−1^ arose from –CH_2_– stretching. Strong absorption in the 1000–1100 cm^−1^ region is mainly assigned to C–O–C and C–O stretching vibrations in cellulose and hemicellulose [32]. In addition, absorption features attributable to Fe–OH (≈1053 cm^−1^) and Mn–O/Mg–O lattice vibrations (400–600 cm^−1^) suggest the coexistence of organic functional groups and inorganic metal oxides on the FMM-BC surface [33].

XRD patterns (Figure 2b) further support this hybrid structure. Broad reflections in the 2*θ* range of 10–40°, centered near 22°, indicate the presence of amorphous or weakly ordered carbon phases typical of partially carbonized biomass. Meanwhile, weak diffraction peaks at approximately 35°, 41°, and 60° correspond to Fe, Mn, and Mg containing oxide phases, including poorly crystalline FeO(OH) [34,35]. Such low-crystallinity metal oxides are known to provide abundant reactive surface sites for arsenic adsorption and co-precipitation, despite their limited thermodynamic stability under prolonged reducing conditions.

XPS analysis (Figure S3) further supports these findings. Distinct Fe 2p and Mn 2p peaks were observed in the FMM-BC spectrum, confirming the incorporation of these elements. Notably, the O 1s spectrum of FMM-BC exhibited a new peak at 529.98 eV, corresponding to metal-bound oxygen (M–O, where M = Fe, Mn, Mg), which confirms the integration of metal oxides [36]. Deconvolution of the Fe 2p spectrum revealed peaks at 713.43, 711.11, and 710.18 eV, attributable to Fe(III) species (FeOOH/Fe_2_O_3_) and some Fe(II) (Fe_3_O_4_/FeO) [36]. Similarly, Mn 2p peaks at 642.29 and 641.04 eV indicated the coexistence of Mn(III) and Mn(II). A Mg 1s binding energy at 1304.67 eV further confirmed the presence of MgO [19]. Among the detectable metal oxides on the surface, iron oxides account for approximately 44.34% and manganese oxides account for about 51.16% of the total. This indicates that Fe(III) phases predominantly govern As(V) adsorption through inner-sphere complexation, while Mn oxides participate mainly in redox-mediated oxidation of As(III) [37,38].

These structural features underpin the high arsenic (As) immobilization capacity of FMM-BC. Meanwhile, SEM also revealed the morphological characteristics of FMM-BC (Figure S4). Surface functional groups (–OH, –COOH, –C=O) and metal hydroxyls (>M–OH) act as reactive coordination sites, facilitating As complexation and the formation of stable species such as As–O, COO–As, and Fe/Mn/Mg–O–As. Under typical soil pH conditions, surface metal hydroxides (>M–OH^2+^) are positively charged, enabling electrostatic attraction of negatively charged As(V) oxyanions via outer-sphere complexation [35]. More importantly, ligand exchange between As(V) and metal hydroxyl groups (>M–OH) leads to the formation of stable inner-sphere complexes [14,19,39]. The reaction process is shown in Equations (1)–(4):

(1) $$>M{\mathrm{OH}}_{2}^{+}+H_{2}\mathrm{As}O_{4}^{-}↔\mathrm{MO}H_{2}{+H}_{2}\mathrm{As}O_{4}^{-}$$

(2) $$>M–\mathrm{OH}+H_{2}\mathrm{As}O_{4}^{-}\to s–\mathrm{OHAsO}{\left(\mathrm{OH}\right)}_{2}^{-}+H^{+}$$

(3) $$2>M{\mathrm{OH}}_{2}^{+}+H_{2}\mathrm{As}O_{4}^{-}↔>\mathrm{MOAsO}({\mathrm{OH}}_{2})+H_{2}O$$

(4) $$>R–\mathrm{CO}O^{-}+H^{+}+H_{2}\mathrm{As}O_{4}^{-}↔R–H_{2}\mathrm{As}O_{4}+\mathrm{HCO}O^{-}$$

### 3.2. Effects of FMM-BC on Soil Properties

#### 3.2.1. pH

**Figure 3:**
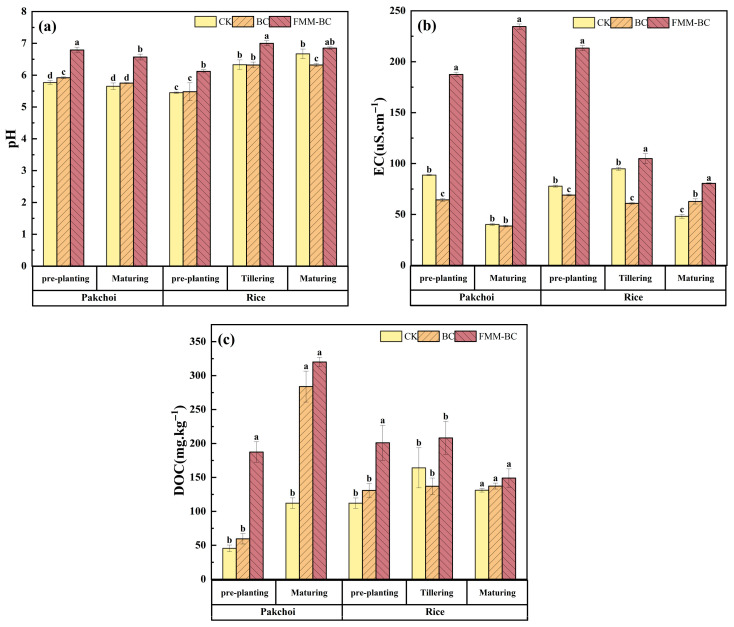
Effects of BC and FMM-BC on (**a**) soil pH, (**b**) soil EC, and (**c**) soil Dissolved Organic Carbon (DOC) content. The letters a, b, c, and d indicate significant differences between treatments, *p* < 0.05.

Soil pH is a key factor regulating the forms and bioavailability of heavy metals. As shown in Figure 3a, application of BC and FMM-BC resulted in distinct pH responses throughout the pakchoi–rice rotation. Prior to planting, soil pH increased from 5.77 in the control (CK) to 5.92 and 6.79 following BC and FMM-BC application, respectively, indicating a stronger alkalizing effect of the ternary-modified material. During pakchoi maturation, soil pH declined slightly in all treatments, which is consistent with rhizosphere acidification induced by root exudation and microbial activity [40,41,42]. Nevertheless, pH values under FMM-BC remained consistently higher than those under BC and CK, suggesting that the buffering capacity introduced by Fe/Mn/Mg-containing phases partially counteracted acidification processes [9].

Following the transition to flooded rice cultivation, soil pH increased across all treatments, reflecting the reductive dissolution of Fe(III) minerals and associated proton consumption under anaerobic conditions [9,16]. Irrigation-drainage cycles drive dissolution and re-precipitation of Fe/Mn (oxyhydr)oxides, while microbial processes (Fe/Mn-reducing bacteria, As-reducing/oxidizing and methylating communities) mediate redox and methylation reactions [40,41,42]. Notably, although FMM-BC maintained a higher absolute pH throughout the experiment, the magnitude of pH increase during rice growth was smaller than that observed in CK. This pattern suggests that Fe/Mn/Mg oxides associated with FMM-BC may have moderated pH fluctuations by undergoing coupled redox and hydrolysis reactions rather than acting solely as alkaline ash inputs [14,40].

#### 3.2.2. EC

Soil electrical conductivity (EC) is an integrated indicator reflecting the total concentration of soluble ions and mineral salts in the soil solution, representing ion migration activity [1]. As shown in Figure 3b, EC increased markedly following the application of FMM-BC compared with the control (CK) and BC treatments during both the pakchoi and rice cultivation periods. Although EC gradually declined with time, it remained consistently higher than CK throughout the experiment.

After the application of FMM-BC, soil EC increased significantly during the first year of rapeseed cultivation and the early stage of rice growth, reaching a peak of 234.5 μS∙cm^−1^. This likely resulted from the release of soluble Fe, Mn, Mg, K, and P, thereby elevating EC values [43,44]. These ions may persist despite post-synthesis washing and contribute substantially to soil ionic strength. However, EC declined during the later stages of rice growth as nutrients were absorbed by plants and Fe/Mn/Mg oxides or hydroxides aged and re-precipitated [45].

#### 3.2.3. DOC

Dissolved organic carbon (DOC) is a key factor influencing the solubility and mobility of trace elements, including heavy metals, in soil. Organic ligands in DOC can increase the solubility of these elements by forming soluble metal–organic complexes, but they may also influence their immobilization through other mechanisms [46]. As shown in Figure 3c, soil DOC concentrations differed substantially among treatments and growth stages. During the pakchoi cultivation period, soil DOC under the FMM-BC treatment increased rapidly, reaching a value 111.79 mg∙kg^−1^ higher than that of CK. This pattern suggests that FMM-BC released a considerable amount of labile organic carbon into the soil solution, which is consistent with the low-temperature pyrolysis of the biomass and the presence of partially decomposed organic components.

During the rice-growing period, DOC concentrations generally decreased across treatments, particularly during the tillering stage. This phenomenon may be attributed to the tillering stage being a critical developmental phase for rice, during which a large amount of DOC is produced due to intense microbial activity. As time progresses and soil microorganisms proliferate, they consume substantial amounts of DOC for metabolic activities, leading to a reduction in DOC content during this stage [47].

### 3.3. Effects of FMM-BC on Arsenic Availability and Fractionation

#### 3.3.1. Water-Soluble and Bioavailable Arsenic

**Figure 4:**
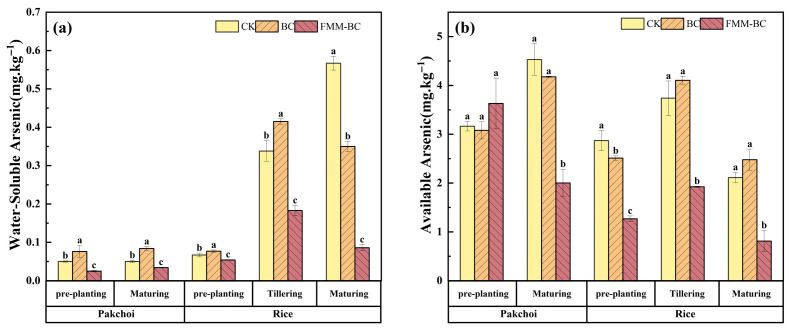
Effects of BC and FMM-BC treatments on (**a**) water-soluble arsenic content and (**b**) available arsenic content in soil at different growth stages. The letters a, b, c indicate significant differences between treatments, *p* < 0.05.

Water-soluble and bioavailable arsenic are considered the most relevant fractions governing short-term arsenic mobility and plant uptake in soil systems [14,48]. As shown in Figure 4a,b, application of FMM-BC resulted in a marked reduction in both water-soluble As and NaHCO_3_-extractable As compared with the control (CK) and BC treatments throughout the pakchoi–rice rotation.

During the pakchoi growth period, soil water-soluble As content in the FMM-BC treatment decreased by more than 40% relative to CK, while soil available As content showed a comparable declining trend, decreasing by 44.84%. In contrast, BC treatment led to only a marginal reduction, at certain stages, a slight increase in available As. This suggests that low-temperature biochar alone was insufficient to immobilize arsenic effectively and may have released labile organic components that enhanced As mobility. The superior performance of FMM-BC can be attributed primarily to the introduction of Fe, Mn, and Mg-bearing mineral phases, which provide additional reactive surfaces capable of binding arsenic. Under the slightly acidic to near-neutral pH conditions maintained during the experiment, these metal-associated surfaces likely favored the adsorption of arsenate through surface complexation, thereby lowering dissolved and exchangeable arsenic pools.

During the rice tillering stage, compared to pre-rice planting, available arsenic content increased in all treatments (CK +30.27%, BC +63.45%, FMM-BC +51.80%). Under anaerobic conditions, As(V) is reduced to the more mobile As(III), and iron–manganese oxides release the adsorbed or coprecipitated arsenic, collectively leading to a surge in water-soluble arsenic concentration [49]. Nevertheless, soils amended with FMM-BC consistently exhibited significantly lower soluble and available As than CK, indicating that the modified material partially mitigated arsenic release during flooding. This stabilizing effect may reflect the presence of newly formed or regenerated Fe/Mn (hydr)oxides associated with FMM-BC.

#### 3.3.2. Fractionation of as in Soil

**Figure 5:**
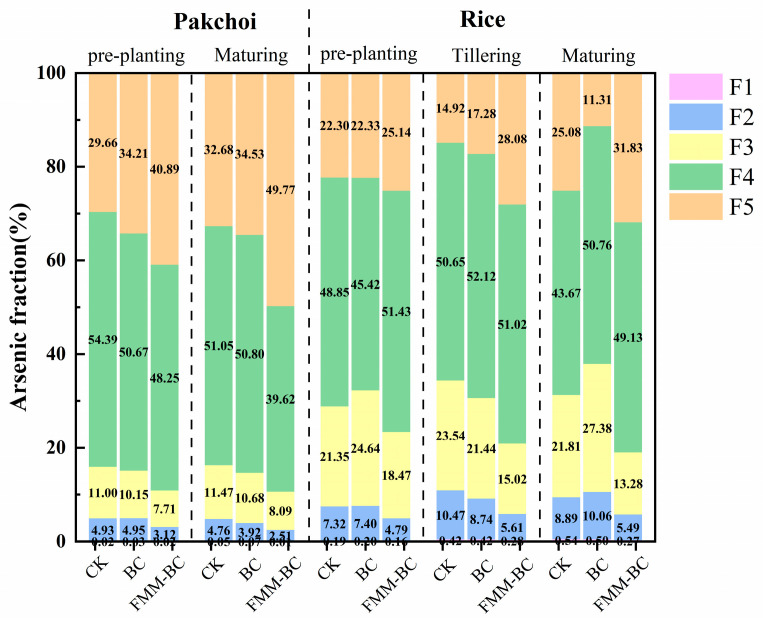
Speciation fractions of arsenic in soil after application of BC and FMM-BC at different growth stages.

The biological toxicity of arsenic in soil is closely related to its bioavailability. To further investigate the impact of passivators on arsenic stability, this study employed the Wenzel sequential extraction method [26] to analyze soil arsenic fractions, classifying them into five forms: exchangeable form (F1), carbonate-bound form (F2), iron–manganese oxide-bound form (F3), organic-bound form (F4), and residual form (F5). Among these, F1 and F2 represent highly active, bioavailable, and unstable forms, whereas F3, F4, and F5 are stable forms with low mobility and bioavailability [50,51]. As shown in Figure 5, sequential extraction revealed a clear shift in arsenic distribution from labile fractions toward more stable forms following FMM-BC application. Specifically, the proportions of water-soluble and exchangeable arsenic (F1–F2) decreased, while the residual fraction (F5) increased significantly compared with CK.

During the rice planting period, the proportions of F1 and F2 remained relatively stable throughout the rice growth stages (pre-planting, tillering, and maturity), with minimal changes across treatments. The F3 content under the FMM-BC treatment was lower than CK, with reductions of 2.88%, 8.52%, and 8.53%, respectively. Notably, during rice maturity, the FMM-BC treatment exhibited a 5.46% increase in F4 and a significant 6.07% increase in F5 compared to CK. In terms of enhancing soil F5 content, the effect of FMM-BC was markedly superior to that of the BC treatment.

#### 3.3.3. Relationship Between Soil Properties, Available Arsenic, Water-Soluble Arsenic and Soil Arsenic Components Was Explored

**Figure 6:**
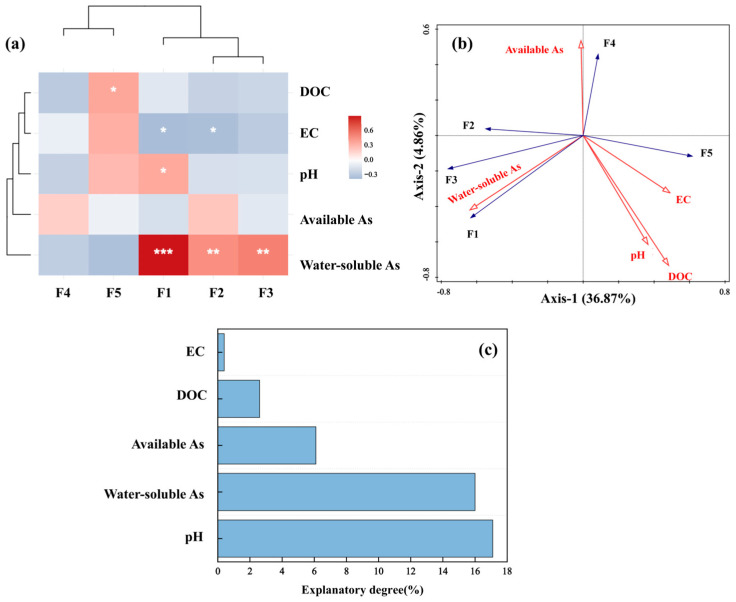
Correlation (**a**) and RDA (**b**,**c**) analyses of soil physicochemical properties, water-soluble arsenic and available arsenic concentrations with arsenic speciation fractions in soil. (Arrows indicate correlation rather than direct causation; interpretation is limited to co-variation among variables; *** indicates that the difference is extremely significant (*p* < 0.001), ** indicates that the difference is highly significant (*p* < 0.01), and * indicates a significant difference (*p* < 0.05)).

To evaluate the effects of FMM-BC on soil properties and arsenic fraction distribution, Spearman correlation analysis (Figure 6a) and redundancy analysis (RDA; Figure 6b) were conducted using soil pH, EC, DOC, available arsenic, water-soluble arsenic, and arsenic fractions (F1–F5). As shown in Figure 6a, water-soluble arsenic was strongly and positively correlated with F1, F2, and F3 (*p* < 0.01 or *p* < 0.001). In contrast, DOC showed a significant positive correlation with the F5 and negative correlations with F1–F4.

The RDA biplot further clarified these relationships. The first two axes explained 36.87% and 9.65% of the total variance, respectively. Soil pH (17.1%, *p* < 0.05) and water-soluble As (16.09%, *p* < 0.05) were identified as significant contributors to As fraction distribution. Soil pH and the more stable fractions (F3–F5) clustered together and were oriented opposite to water-soluble As and labile fractions (F1–F2), indicating their antagonistic roles in controlling arsenic mobility. Under neutral to slightly alkaline aerobic conditions, arsenic mainly exists as negatively charged arsenate species ($H_{2}{\mathrm{AsO}}_{4}^{-}$, ${\mathrm{HAsO}}_{4}^{2-}$), which are readily adsorbed onto Fe (hydr)oxide surfaces.

After two years of pakchoi–rice rotation, soil pH exerted a measurable influence on soluble arsenic, consistent with previous reports that elevated pH can increase available As [52,53]. Meanwhile, the shift in As toward more stable fractions (F3–F5) with increasing pH is likely related to enhanced adsorption of As(V) by Fe (hydr)oxides, promoting immobilization into less mobile forms [54]. Soil EC was negatively correlated with F1 and F2 (*p* < 0.05), suggesting that changes in ionic strength affected As adsorption equilibria [55,56]. However, enhanced As stabilization following FMM-BC application was more closely associated with Fe/Mn oxide-derived adsorption and co-precipitation sites than with EC increases alone [10].

FMM-BC increased soil DOC content, reaching up to 320 mg∙kg^−1^. Increased DOC content may stimulate microbial activity and Fe(III) reduction, thereby influencing arsenic speciation under flooded conditions [57]. Although prolonged reduction can destabilize Fe–As associations and release As(III) [22], the concurrent increase in F5 suggests that DOC also participated in As stabilization via co-precipitation and encapsulation with Fe/Mn/Mg oxides. Previous studies have shown that such organic–mineral complexes (e.g., Fe–C or Fe–As–C) exhibit low solubility and high structural stability, effectively reducing arsenic mobility [58,59,60]. The simultaneous increase in F5 fraction up to 31.83%, decrease in Fe/Mn-extractable forms, and increased pH collectively indicate that re-precipitation of Fe/Mn/Mg oxides and DOC encapsulation are the dominant mechanisms of As stabilization.

Finally, water-soluble As speciation is primarily governed by redox potential and pH. As(III) dominates under anaerobic conditions, whereas As(V) prevails under aerobic conditions and is more strongly retained by iron oxides [49,61]. The Fe/Mn/Mg oxides loaded onto FMM-BC slow down dissolution processes and provide additional high-affinity adsorption sites [49,62].

### 3.4. Effects of BC and FMM-BC on Arsenic Uptake and Internal Transport in Pakchoi and Rice

#### 3.4.1. Content of as in Pakchoi and Rice

**Figure 7:**
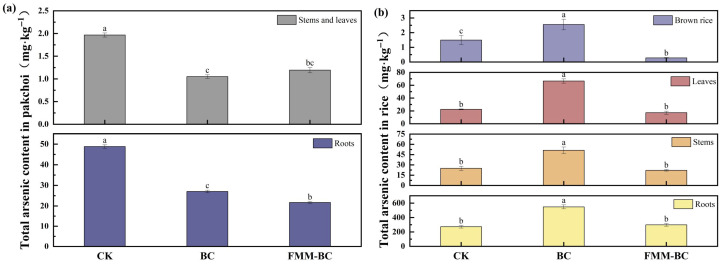
Effects of BC and FMM-BC on arsenic content in pakchoi (**a**) and rice (**b**). The letters a, b, c indicate significant differences between treatments, *p* < 0.05.

The total arsenic content in different tissues (roots, stems, leaves, and brown rice) of pakchoi and rice showed variation after the application of BC and FMM-BC to arsenic-contaminated soil (Figure 7). During the pakchoi growth period, roots were the primary As sink, consistent with previous reports indicating that up to 90–95% of plant As is retained in root tissues [2]. Relative to the control (CK), BC and FMM-BC treatments reduced root As concentrations by 44.63% and 55.72%, respectively. Correspondingly, As concentrations in pakchoi stems and leaves decreased substantially, with reductions of 46.66% and 56.26% under FMM-BC treatment compared with CK (1.966 mg kg^−1^).

These results indicate that both BC and FMM-BC effectively suppressed As uptake during the aerobic pakchoi stage, with FMM-BC showing a consistently stronger inhibitory effect. This enhanced performance is consistent with the greater capacity of Fe/Mn/Mg-modified materials to immobilize soil As and reduce its availability to plant roots. In contrast, As accumulation patterns during the rice growth period differed markedly, reflecting the strongly reducing conditions imposed by flooded cultivation. Under BC treatment, root As concentration increased sharply by 101.36%, reaching 547.58 mg kg^−1^, suggesting that unmodified biochar was insufficient to counteract As mobilization under anaerobic conditions. Moreover, BC application significantly increased As concentrations in rice stems, leaves, and brown rice by 105.68%, 196.28%, and 70.94%, respectively, indicating enhanced translocation of As from roots to aboveground tissues.

By comparison, FMM-BC treatment effectively constrained As accumulation across rice tissues. Root As increased only slightly (9.63%, 198.12 mg kg^−1^), while As concentrations in stems, leaves, and brown rice decreased by 11.79%, 23.33%, and 81.28%, respectively. Importantly, As concentration in brown rice under FMM-BC treatment was maintained at 0.27 mg kg^−1^, well below the Chinese food safety limit of 0.35 mg kg^−1^ (GB 2762-2022) [63]. These contrasting responses highlight the critical role of Fe/Mn/Mg modification in maintaining As immobilization under flooded conditions.

#### 3.4.2. Arsenic Translocation and Bioconcentration Within Rice Plants

To further elucidate the internal transport behavior of As in rice, transfer factors (TF) and bioconcentration factors (BCF) were calculated (Table 1, Figure S2). Across all treatments, As transfer followed the general sequence TF_stem–leaf_ > TF_stem–root_ > TF_stem–grain_, indicating that As movement from stems to grains is intrinsically restricted relative to vegetative tissues. BC application increased TF_stem–leaf_ by 24.27% compared with CK, while only slightly reducing TF_stem–grain_ (−14.13%). This limited reduction suggests that BC alone exerted minimal control over As translocation to edible tissues. In contrast, FMM-BC significantly reduced TF values across all transport pathways, particularly TF stem–grain, which declined by 78.64% relative to CK. This pronounced decrease indicates that FMM-BC effectively restricted upward As movement from stems into rice grains.

Consistent trends were observed for BCF. BC treatment markedly increased BCF values in rice roots, stems, leaves, and brown rice, reflecting enhanced As accumulation capacity under reducing conditions. Conversely, FMM-BC reduced BCF values in aboveground organs by 11.79–81.27%, while maintaining root BCF at levels comparable to CK. These results indicate that FMM-BC not only limits As uptake at the soil–root interface but also suppresses its internal redistribution within the plant.

#### 3.4.3. Mechanistic Interpretation of Reduced as Accumulation Under FMM-BC Treatment

**Figure 8:**
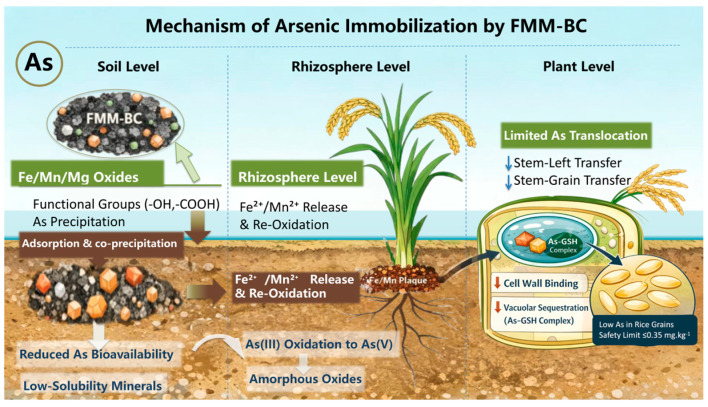
The proposed mechanism of arsenic immobilization by FMM-BC. The downward arrow indicates the transfer coefficient from stem to leaf and from stem to grain decreases.

The observed reductions in As uptake and translocation under FMM-BC treatment can be interpreted as the combined outcome of soil-level stabilization and plant-level transport restriction. The proposed possible mechanism is illustrated in Figure 8. At the soil interface, FMM-BC provides abundant reactive sites associated with oxygen-containing functional groups and Fe/Mn/Mg-bearing phases, which enable strong complexation with As(III) and electrostatic attraction of As(V) under typical soil pH conditions [10]. FMM-BC application also drives a pronounced shift in arsenic speciation from labile to stable fractions, primarily through the formation of amorphous and crystalline metal–arsenate complexes (e.g., Fe/Mn/Mg arsenates) via specific adsorption and co-precipitation with biochar-associated oxides [34,62]. However, the timescale of this stabilization effect remains uncertain. long-term or cyclic flooding-drainage experiments are necessary to evaluate the durability and potential degradation rate of FMM-BC performance.

During the aerobic growth stage, the soil remains in an oxidizing state, which promotes the precipitation of Fe/Mn oxides and the adsorption of As, thereby strengthening the short-term stabilization effect [48]. However, when the soil is subsequently flooded under reducing conditions, these newly formed amorphous or poorly crystalline oxides may be rapidly reduced and dissolved, resulting in the remobilization of arsenic [17]. In contrast, FMM-BC likely functions as a potentially sustained source of Fe^2+^, Mn^2+^, and Mg^2+^ that can regenerate amorphous metal (hydr)oxides during drainage or within rhizosphere microzones, thereby maintaining As immobilization under fluctuating redox conditions [49,64].

At the plant level, the strong reduction in FMM-BC effectively restricts As translocation beyond the stem. Previous studies have shown that Fe-modified biochar promotes the formation of Fe plaques on root surfaces and enhances Fe accumulation in the cell wall, thereby enhancing As sequestration and limiting As(III) transport through the xylem [49,65]. In addition, intracellular sequestration of As in root vacuoles further limits its mobility toward grains [66,67].

Overall, FMM-BC with low carbonization temperature offers low-cost, mineral–organic dual functionality, and multi-pathway regulatory capacity. By simultaneously reducing soil arsenic bioavailability and inhibiting arsenic transport in rice, its efficacy surpasses that of unmodified biochar. Future studies should quantitatively analyze root-surface Fe plaque formation and plant antioxidant enzyme activity to further elucidate the mechanisms of rhizospheric As sequestration and transport regulation.

## 4. Conclusions

This study evaluated the remediation performance of Fe, Mn, and Mg co-modified biochar (FMM-BC) in arsenic-contaminated paddy soil using a controlled pot experiment. The results indicate that ternary modification provided additional reactive surfaces and improved arsenic immobilization compared with unmodified biomass. FMM-BC application reduced arsenic bioavailability by more than 50%, accompanied by a clear shift of arsenic from labile fractions (F1–F2) to more stable Fe/Mn-associated and residual fractions (F3–F5). FMM-BC significantly lowered arsenic accumulation in both pakchoi and rice, with arsenic content in rice stems, leaves, and brown rice decreasing to 1.94 mg·kg^−1^, 5.24 mg·kg^−1^, and 1.21 mg·kg^−1^, respectively. The arsenic content in brown rice remained below the food safety threshold (0.35 mg·kg^−1^). The reduction of arsenic uptake by rice can be attributed to three complementary mechanisms: (i) ligand exchange forming inner-sphere complexes between arsenic oxyanions and ≡Fe–OH/≡Mn–OH surface sites; (ii) co-precipitation with Fe/Mn/Mg (hydr)oxides that incorporates arsenic into insoluble phases; and (iii) redox cycling of Fe and Mn, whereby Fe(III) and Mn(III,IV) oxidize As(III) to the less mobile As(V). Derived from low-cost biomass and common metal salts, FMM BC also supplies Fe, Mn, and Mg as micronutrients, which may contribute to its performance in the soil–plant system. Future studies should focus on field scale validation, long term arsenic stability under fluctuating redox conditions, and the potential release of co-introduced elements to better assess the agronomic feasibility and environmental safety of FMM-BC.

## Figures and Tables

**Table 1:** Changes in TF and BCFs of arsenic in rice grown in soil treated with BC and FMM-BC.

Treatment	TF_Stem/Root_	TF_Leaf/Stem_	TF_Brown rice/Stem_	BCF_Root_	BCF_Stem_	BCF_Leaf_	BCF_Brown rice_
CK	0.09 ± 0.01 ^a^	0.91 ± 0.11 ^a^	0.06 ± 0.01 ^a^	1.51 ± 0.10 ^b^	0.14 ± 0.02 ^a^	0.13 ± 0.00 ^b^	0.01 ± 0.00 ^b^
BC	0.09 ± 0.02 ^a^	1.13 ± 0.35 ^a^	0.05 ± 0.01 ^a^	3.05 ± 0.26 ^a^	0.29 ± 0.07 ^a^	0.31 ± 0.08 ^a^	0.01 ± 0.00 ^a^
FMM-BC	0.07 ± 0.00 ^a^	0.78 ± 0.12 ^a^	0.01 ± 0.00 ^b^	1.66 ± 0.10 ^b^	0.12 ± 0.01 ^b^	0.10 ± 0.01 ^b^	0.00 ± 0.01 ^c^

Notes: Results are expressed as the arithmetic mean ± standard deviation. Different lowercase letters in the same column indicate significant differences (*p* < 0.05).

## Data Availability

The raw data supporting the conclusions of this article will be made available by the authors on request.

## Supplementary Materials

The following supporting information can be downloaded at: https://www.mdpi.com/article/10.3390/toxics14020112/s1, Text S1. Preparation of sugarcane bagasse BC and FMM-BC. Text S2. Response Surface Optimization and Model Analysis. Text S3. Determination of the optimal amendment rate. Text S4. Calculation of Field Capacity. Text S5. The pot design is account details. Table S1. Basic physical and chemical properties of the soil. Table S2. Three-factor three-level factor test table. Table S3. Design and experimental results of RSM. Table S4. Analysis of variance of available arsenic contents (Y_1_) regression model. Table S5. Analysis of variance of biochar yield (Y_2_) regression model. Figure S1. Determination of the optimal amendment rate of BC and FMM-BC. Figure S2. (a) Correlation between arsenic content and arsenic transfer coefficient (TF) in different tissues of rice. (b) Correlation between arsenic content and arsenic bioconcentration factor (BCF) in different tissues of rice. Figure S3. XPS spectra of pristine BC and FMM-BC. Figure S4. SEM of BC and FMM-BC.
