# Supplementary material for: Iron–Manganese–Magnesium Co-Modified Biochar Reduces Arsenic Mobility and Accumulation in a Pakchoi–Rice Rotation System
Source: Toxics. 2026 Jan 24;14(2):112. doi: 10.3390/toxics14020112 (PMC12944937; doi:10.3390/toxics14020112)
Supplement: Supplementary file 1 [file toxics-14-00112-s001.zip › toxics-4081825-supplementary.pdf]

Supplementary Materials

Iron–Manganese–Magnesium Co-Modified Biochar Reduces Arsenic Mobility and Accumulation in a Pakchoi–Rice Rotation System

Jingnan Zhang [1], Meina Liang [1,2,3,*], Mushi Qiao [1], Qing Zhang [1,2,3], Xuehong Zhang [1,2,3] and Dunqiu Wang [1,2,3]

[1] College of Environmental Science and Engineering, Guilin University of Technology, Guilin 541004, China
[2] Guangxi Key Laboratory of Environmental Pollution Control Theory and Technology, Guilin University of Technology, Guilin 541004, China
[3] Engineering Research Center of Watershed Protection and Green Development, Guilin University of Technology, Guilin, 541006, China
* Correspondence: Meina Liang (Email: liangmeina@glut.edu.cn)

Text S1 Preparation of sugarcane bagasse BC and FMM-BC

1. Preparation of sugarcane bagasse BC

Sugarcane bagasse passed through a 40-mesh sieve was placed in a ceramic boat and pyrolyzed in a tubular furnace under a nitrogen atmosphere. The temperature was increased to 300 °C at a heating rate of 5 $°C·min^{-1}$ and maintained for 1 h. After cooling to room temperature, the product was ground and passed through a 100-mesh sieve to obtain the raw sugarcane bagasse biochar, denoted as BC.

2. Preparation of FMM-BC

A total of 30 g of solid KOH was dissolved in 300 mL of ultrapure water, and the solution was added to 50.00 g of sugarcane bagasse placed in a 1000 mL beaker. The mixture was soaked for 24 h. Subsequently, 100 mL of 0.5 $mol·L^{-1}$ $MnSO_4$ solution, 100 mL of 0·5 $mol.L^{-1}$ $MgCl_2$ solution, and 200 mL of 1 $mol·L^{-1}$ $Fe(NO_3)_3$ solution were added to the impregnated bagasse. The suspension was stirred at 400 $r·min^{-1}$ for 30 min at 25 °C using a magnetic stirrer, after which the pH was adjusted to 12.0 with 5 $mol·L^{-1}$ KOH solution. After being stirred for an additional 30 min and allowed to stand for 24 h, the mixture was transferred to a 2000 mL beaker, sequentially washed twice with deionized water and twice with anhydrous ethanol until the filtrate remained neutral, and then filtered. The filter cake was dried in an oven at 80 °C and subsequently pyrolyzed in a tubular furnace. The temperature was raised to 300 °C at a heating rate of 5 $°C·min^{-1}$ and held for 1 h. After cooling to room temperature, the product was sieved through a 100-mesh sieve to obtain the modified biochar, denoted as FMM-BC.

Text S2 Response Surface Optimization and Model Analysis

Quadratic polynomial regression models were developed to describe the relationships between the independent variables and the response variables, namely soil bioavailable arsenic content (Y_1) and biochar yield (Y_2), as expressed by the following coded equations:

$$Y_1=1.69+0.2143A-0.1410B+0.0528C+0.4537AB+0.0978AC+0.1278BC +0.54958A^2+0.05108B^2-0.4865C^2 \quad (1)$$

$$Y_2=50.10-2.30A+0.3363B-15.48C+0.3925AB-0.2000AC+0.8550BC -2.03A^2+1.18B^2+9.24C^2 \quad (2)$$

For Y_1, the regression model exhibited a high level of statistical significance ($F = 21.6$, $P < 0.01$), while the lack-of-fit test was not significant ($P = 0.9358 > 0.05$), indicating that the model adequately described the experimental data. The coefficient of determination (R^2) was 0.9652, and the adjusted R^2 was 0.9634, demonstrating excellent model fitting and predictive capability. Moreover, the difference between the adjusted R^2 and predicted R^2 was less than 0.2, the adequate precision exceeded 4, and the coefficient of variation (C.V.%) was below 10%, confirming the robustness and reliability of the model.

Analysis of individual model terms revealed that A (activator-to-bagasse mass ratio), AB, A^2, and C^2 were highly significant ($p < 0.01$), while B (Mn^{2+}:Mg^{2+} molar ratio) was significant at $P < 0.05$. In contrast, the linear term of pyrolysis temperature (C) was not significant ($p > 0.05$). The relative influence of the factors on soil bioavailable arsenic followed the order: Mn^{2+}:Mg^{2+} molar ratio > activator-to-bagasse mass ratio > pyrolysis temperature. After excluding non-significant terms, the simplified regression equation for Y_1 was obtained as:

$$Y_1=1.6872+0.21425A-0.141B+0.45375AB+0.549525A^2-0.486475C^2 \quad (3)$$

The yield of Fe/Mn/Mg-modified sugarcane bagasse biochar ranged from 37.83% to 77.40% (Table S3). Higher yields were generally achieved under relatively lower pyrolysis temperatures and lower activator-to-bagasse mass ratios.

For Y_2, the regression model was also highly significant ($p < 0.01$), with a non-significant lack-of-fit ($p > 0.05$). The values of R^2 and adjusted R^2 were very close, and the difference between adjusted R^2 and predicted R^2 was less than 0.2. In addition, adequate precision exceeded 4 and C.V.% was below 10%, indicating satisfactory model reliability.

Among the individual factors, A was significant at $p < 0.05$, while C and C^2 were highly significant ($p < 0.01$), demonstrating that pyrolysis temperature exerted the strongest influence on biochar yield. The Mn^{2+}:Mg^{2+} molar ratio (B) showed no significant effect ($p > 0.05$). The order of influence on biochar yield was: pyrolysis temperature > activator-to-bagasse mass ratio > Mn^{2+}:Mg^{2+} molar ratio. After removing non-significant terms, the simplified regression equation for Y_2 was expressed as:

$$Y_2=50.10-2.30A-15.48C+9.24C^2 \quad (4)$$

Text S3 Determination of the optimal amendment rate

Fifty grams of the arsenic-contaminated soil was placed in a 100 mL polyethylene bottle. BC and FMM-BC were incorporated into the soil at mass ratios of 0.5%, 1.0%, 2.0%, 4.0%, 5.0%, 6.0%, and 7.0%, respectively. A control (CK) without biochar addition was also included. Each treatment was performed in triplicate. Deionized water was added to achieve 70% of the soil's field water-holding capacity. The bottles were then covered with perforated caps and incubated in a constant-temperature and humidity chamber at 25 ±1 °C. The water content was maintained by weighing every 2 – 4 days and adjusting with deionized water (maximum deviation ± 0.9%). Soil samples were collected on the 10th day of incubation. After air-drying, the samples were sieved to < 0.25 mm, homogenized using the quartering method, and stored in sealed polyethylene bags for subsequent analysis of available arsenic.

According to Figure S1, the addition of BC exhibited limited effectiveness in immobilizing available arsenic in soil. In most cases, BC application even increased the concentration of available As, except at addition rates of 4.0%, 6.0%, and 7.0%, which slightly reduced available As by 4.79%, 6.32%, and 18.00%, respectively. In contrast, FMM-BC consistently decreased available As across all addition levels, and the immobilization efficiency improved with increasing amendment rates. The reductions reached 26.76%, 30.96%, 34.54%, and 36.55% at 4.0%, 5.0%, 6.0%, and 7.0% application levels, respectively. Therefore, a 4.0% amendment rate was selected for subsequent pot experiments based on its effectiveness and practical applicability.

Text S4 Calculation of Field Capacity

Field capacity (FC) refers to the maximum capillary water content that soil can retain after excess gravitational water has drained away following irrigation or rainfall. It is usually expressed as a percentage of the soil's water content by weight. In this study, 70% of the field capacity was set as the target soil moisture, meaning that the actual soil water content was maintained at 70% of FC. The field capacity of the tested soil was determined to be 30%, and the dry soil mass used in the experiment was 4 kg.

Thus, the target moisture content was calculated as:

$$\text{Target moisture} = 30\% \times 70\% = 21\%.$$

As described in Text S1, the initial soil moisture content was 16.02%. However, before the pot experiment, the soil was air-dried to a moisture content of 0%. The required amount of water was therefore calculated as:

$$W_{water} = 4\ \text{kg} \times 21\% = 0.85\ \text{kg}.$$

Accordingly, 0.85 kg of deionized water was added to each container to achieve 70% of the field capacity.

Text S5 The pot design is account details:

1. Pot material (plastic flower pot);
2. Pot dimensions (length 25.5cm, width 16.5 cm, height 15cm);
3. Soil weight (4 kg);
4. Drainage conditions (alternation of wetting and drying);
5. Number of replicates per treatment (n = 3);
6. Randomization procedure;
7. Spatial arrangement to avoid shading.

Table S1. Basic physical and chemical properties of the soil.

Test index	Value	Methods
Soil type	paddy soil	Soil texture triangle (USDA, 1951).
pH	5.96	pH meter (E-201-C, China, Shanghai)
DOC ($mg{\cdot}kg^{-1}$)	152.55	TOC-1020A organic carbon analyzer (Multi N/C 3100, Germany, Analytik Jena)
Water content (%)	16.02	Gravimetric method，HJ 613-2011
EC ($uS{\cdot}cm^{-1}$)	26.7	Conductivity meter (DDS-801, China, Shanghai)
SOM ($g{\cdot}kg^{-1}$)	45.92	Potassium dichromate-sulfuric acid oxidation method
Total Fe ($mg{\cdot}kg^{-1}$)	61608.8	ICP-OES (Optima 7000DV, Perkin Elmer, American)
Total Mn ($mg{\cdot}kg^{-1}$)	3100.1	ICP-OES (Optima 7000DV, Perkin Elmer, American)
Total As ($mg{\cdot}kg^{-1}$)	179.76	AFS, GB/T 22105.2-2008, China, Beijing
Available As ($mg{\cdot}kg^{-1}$)	3.10	AFS, GB/T 22105.2-2008, China, Beijing

Table S2. Three-factor three-level factor test table.

Factor	Level		
	-1	0	1
(A) Mass ratio of activator to bagasse	0.4	0.6	0.8
(B) Molar ratio of Mn^{2+}: Mg^{2+}	0.5	1.25	2.0
(C) Pyrolysis temperature（℃）	300	500	700

Table S3. Design and experimental results of RSM.

Number	Mass ratio of activator to bagasse	Molar ratio of Mn^{2+}: Mg^{2+}	Pyrolysis temperature（℃）	Available arsenic contents ($mg \cdot kg^{-1}$)	Yield(%)
1	0.4	0.5	500	2.629	49.22
2	0.8	0.5	500	2.166	47.55
3	0.4	2	500	1.502	50.14
4	0.8	2	500	2.854	50.04
5	0.4	1.25	300	1.568	76.37
6	0.8	1.25	300	1.785	68.45
7	0.4	1.25	700	1.52	46.55
8	0.8	1.25	700	2.128	37.83
9	0.6	0.5	300	1.52	77.40
10	0.6	2	300	0.92	75.33
11	0.6	0.5	700	1.328	43.98
12	0.6	2	700	1.239	45.33
13	0.6	1.25	500	1.626	53.49
14	0.6	1.25	500	1.61	50.34
15	0.6	1.25	500	1.844	49.77
16	0.6	1.25	500	1.884	49.49
17	0.6	1.25	500	1.472	47.39

Table S4. Analysis of variance of available arsenic contents (Y_1) regression model.

Variance source	Quadratic sum	Degree of freedom	Mean square	F	P	Conspicuousness, significance
Model	3.64	9	0.4048	21.6	0.0003	**
A	0.3672	1	0.3672	19.59	0.0031	**
B	0.159	1	0.159	8.49	0.0226	*
C	0.0223	1	0.0223	1.19	0.3119	
AB	0.8236	1	0.8236	43.94	0.0003	**
AC	0.0382	1	0.0382	2.04	0.1963	
BC	0.0653	1	0.0653	3.48	0.1042	
A^2	1.27	1	1.27	67.85	< 0.0001	**
B^2	0.011	1	0.011	0.5849	0.4694	
C^2	0.9965	1	0.9965	53.17	0.0002	**
Residual	0.1312	7	0.0187			
Fictitious term	0.0119	3	0.004	0.1324	0.9358	
Pure error	0.1193	4	0.0298			
Overall error	3.77	16				
R^2=0.9652; R^2Adj=0.9206; R^2Pred=0.9004; Adeq Precision=17.9485; C.V.%=7.86						

Notes: ** indicates that the difference is extremely significant ($p<0.01$), and * indicates a significant difference ($p<0.05$)

Table S5. Analysis of variance of biochar yield (Y_2) regression model.

Variance source	Quadratic sum	Degree of freedom	Mean square	F	P	Conspicuousness, significance
Model	2344.62	9	260.51	36.29	< 0.0001	**
A	42.37	1	42.37	5.9	0.0455	*
B	0.9045	1	0.9045	0.126	0.7331	
C	1917.66	1	1917.66	267.1	< 0.0001	**
AB	0.6162	1	0.6162	0.0858	0.778	
AC	0.16	1	0.16	0.0223	0.8855	
BC	2.92	1	2.92	0.4073	0.5437	
A^2	17.42	1	17.42	2.43	0.1632	
B^2	5.82	1	5.82	0.8107	0.3978	
C^2	359.35	1	359.35	50.05	0.0002	**
Residual	50.26	7	7.18			
Fictitious term	30.88	3	10.29	2.13	0.2398	
Pure error	19.37	4	4.84			
Overall error	2394.88	16				
R^2=0.9790; R^2Adj=0.9520; R^2Pred=0.7810; Adeq Precision=18.0993; C.V.%=4.96						

Notes: ** indicates that the difference is extremely significant ($p<0.01$), and * indicates a significant difference ($p<0.05$)

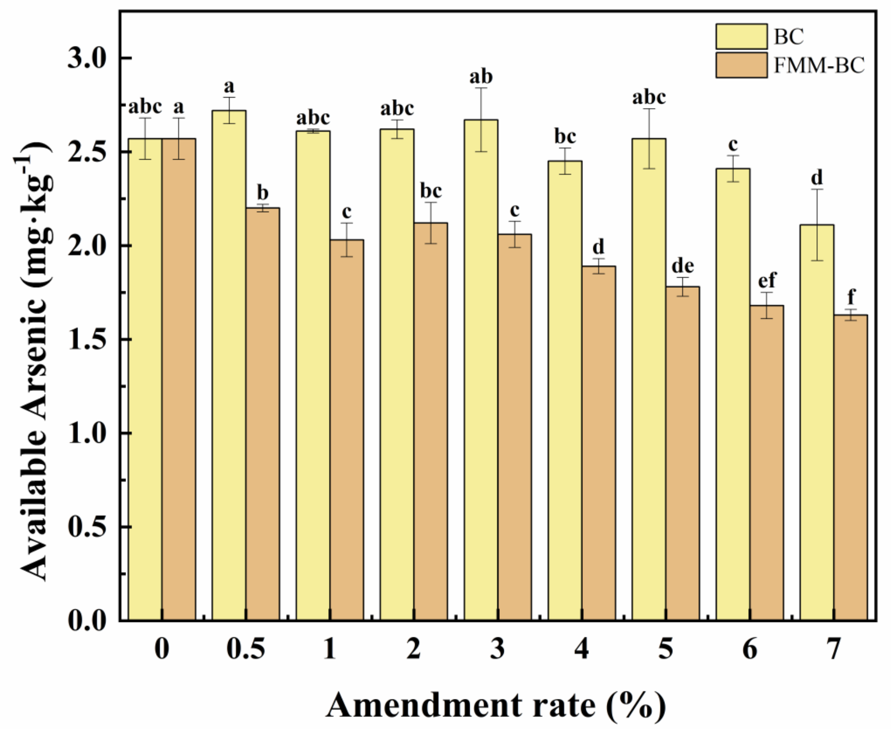

Figure S1. Determination of the optimal amendment rate of BC and FMM-BC.

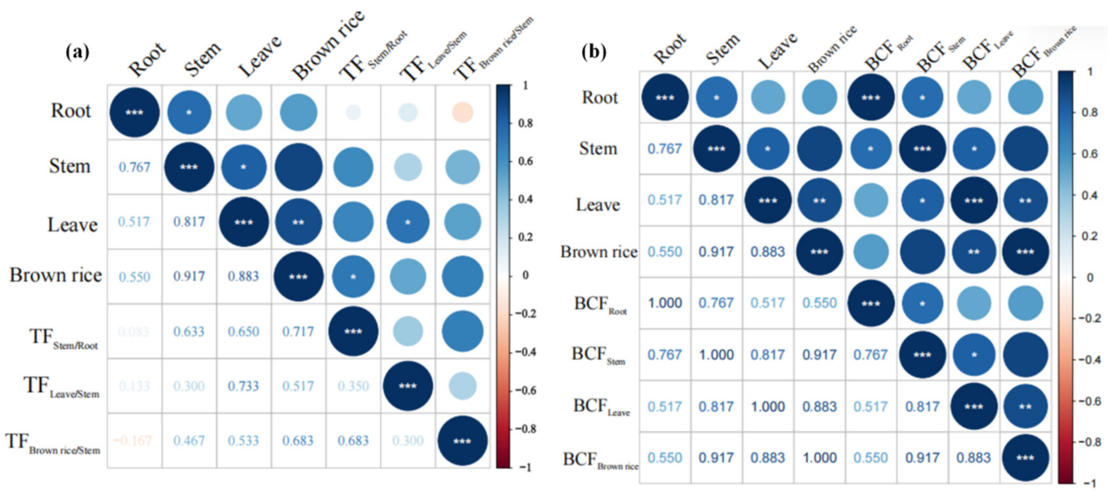

Figure S2. (a) Correlation between arsenic content and arsenic transfer coefficient (TF) in different tissues of rice. (b) Correlation between arsenic content and arsenic bioconcentration factor (BCF) in different tissues of rice.

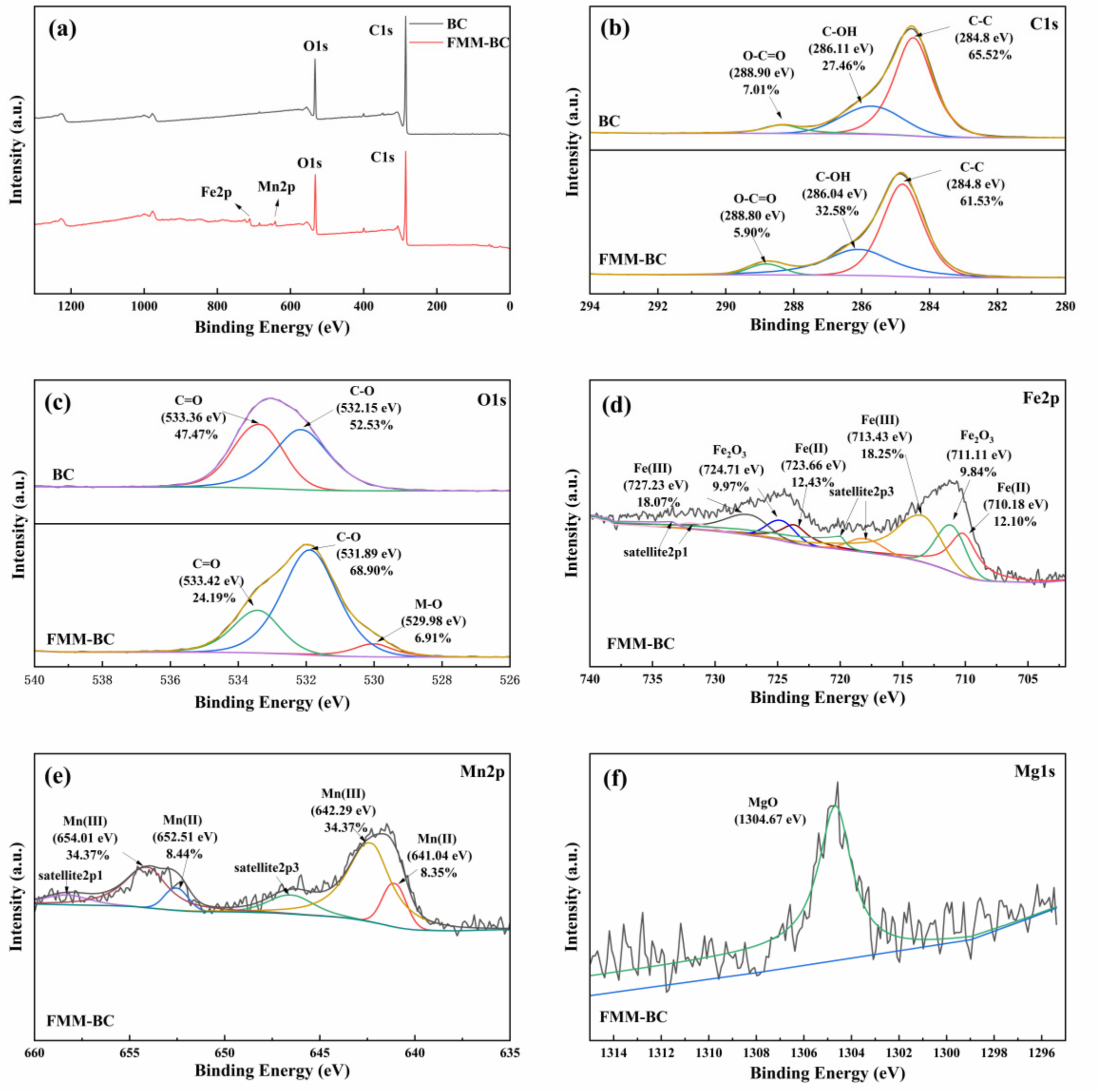

Figure S3. XPS spectra of pristine BC and FMM-BC: (a) XPS wide-scan spectra, C 1s (b), O 1s (c), Fe 2p (d), Mn 2p (e), and Mg 1s (f).

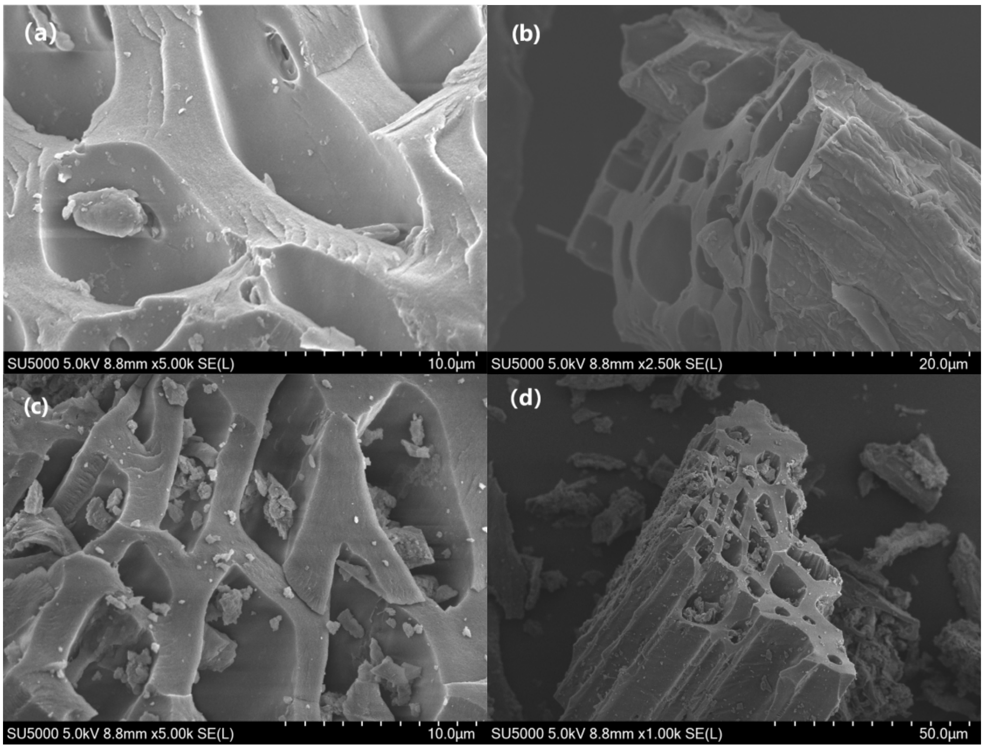

Figure S4. SEM of BC and FMM-BC. (a) and (b): BC; (c) and (d): FMM-BC
